# Rapid endosomal escape of prickly nanodiamonds: implications for gene delivery

**DOI:** 10.1038/srep11661

**Published:** 2015-06-30

**Authors:** Zhiqin Chu, Kaikei Miu, Pingsai Lung, Silu Zhang, Saisai Zhao, Huan-Cheng Chang, Ge Lin, Quan Li

**Affiliations:** 1 Department of Physics, The Chinese University of Hong Kong, Shatin, New Territories, Hong Kong; 2 3rd Institute of Physics, University of Stuttgart, 70569 Stuttgart, Germany; 3 School of Biomedical Sciences, Faculty of Medicine, The Chinese University of Hong Kong, Shatin, New Territories, Hong Kong; 4 Institute of Atomic and Molecular Sciences, Academia Sinica, Taipei 106, Taiwan; 5 The Chinese University of Hong Kong ShenZhen Research Institute, ShenZhen, China

## Abstract

The prickly nanodiamonds easily entered cells via endocytosis followed by unique intracellular translocation characteristics—quick endosomal escape followed by stable residence in cytoplasm. Endosomal membrane rupturing is identified as the major route of nanodiamonds’ escaping the vesicle confinement and to the cytoplasm. Little cytotoxicity is observed to associate with the nanodiamonds’ cytosolic release. Such features enable its application for gene delivery, which requires both effective cellular uptake and cytosolic release of the gene. Taking green fluorescent protein gene as an example, we demonstrate the successful cytosolic delivery and expression of such a gene using the prickly nanodiamonds as carrier.

The superior optical, magnetic properties and biocompatibility of nanodiamonds (NDs) make it a focus of recent research in drug delivery^1^,^2^, bio-imaging^3^,^4^, bio-sensing^5^,^6^ and tissue engineering^7^,^8^. Similar to other nanoparticles (NPs), NDs enter cells via endocytosis, which is an efficient cellular uptake route. Although endocytosis provides a unique opportunity for NPs serving as carriers for macromolecules of low cellular uptake efficiency (e.g., negatively charged DNA and siRNA etc.), effective cytosolic release of the molecules is required in many cases for their proper function^9^. Unfortunately, endocytosis not only results in confinement of NPs in membrane bounded vesicles, i.e., endosomes or lysosomes, inhibiting the cytosolic release can causing possible degradation of macromolecules, but also leads to increased probability of NPs’ cellular excretion via exocytosis^10^.

There are a couple of well-established mechanisms that enable the cytosolic release of the NPs upon endocytosis. Cationic NPs are well known for its disruption of the phosphor lipid membrane due to charge interactions, followed by “proton sponge” effect, leading to osmotic swelling and eventually rupture of the vesicle membrane^11^,^12^. This serves as the most popular method to enable cytosolic release of the NPs—via charge interactions. In addition, specific chemical molecules loaded onto NPs can also damage the bilayer membrane of the vesicle compartments via chemical reactions, and thus cause the cytosolic release of the NPs^13^. Nevertheless, in both cases, toxic events may occur and result in undesired cytotoxicity leading to limited applications. Another issue is the formation of protein corona, once the NPs enter the cells. This makes the NPs’ surface chemistry more complicated and lack of control^14^,^15^. In this regard, an alternative material parameter of NPs is highly desirable to enable their cytosolic release. Most recently, we discovered shape effect of NPs on their intracellular trafficking^16^,^17^. In particular, we found whether the NPs can escape the membrane bounded vesicles upon endocytosis was determined by the circularity of the NPs, i.e., the smaller the circularity (e.g. the sharper the NPs), the easier the escape. However, how would the sharp-edged/cornered NDs escape the vesicles remain puzzling, i.e., they might exit the vesicles by rupturing, or by slipping through the lipid bilayers of vesicle membrane, the biological consequence of which two processes could be very different.

By designing a NDs and SiO_2_ NPs co-feeding experiment in the present work, we showed that the quick escape of NDs (via high pressure high temperature (HPHT) process) from the endosomes was realized by rupturing the endosomal membrane rather than slipping through it. With such unique feature identified, we further utilized the NDs as carriers for genes (plasmid DNA pEGFP-N1) and demonstrated effective plasmid DNA delivery *in vitro*. We showed that easy cellular uptake of NDs and their quick endosomal escape after endocytosis, were the two prerequisites for the effective cytosolic delivery of plasmid DNA. Our findings about NDs as carriers can be generally applied to the delivery of many cell membrane impermeable molecules, such as DNA/siRNA, protein, drug and etc.

## Results and discussions

### The detailed cellular uptake pathway of NDs

**Figure 1:**
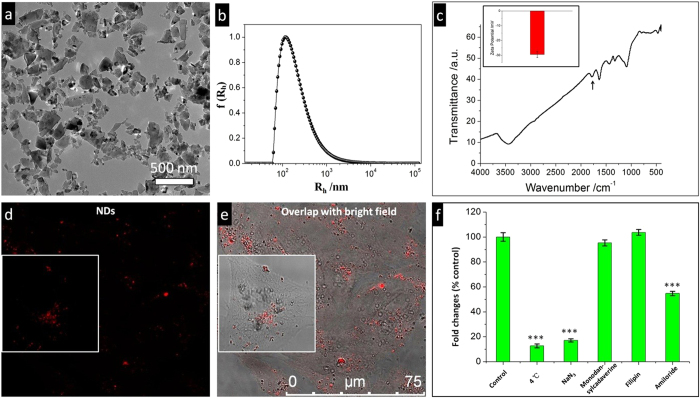
Characterizations of nanodiamonds and their cellular uptake pathways. (**a**) TEM image of nanodiamonds showing their irregular shape with sharp edge and corners. (**b**) Dynamic light scattering (DLS) result of nanodiamonds dispersed in PBS buffer. (**c**) FTIR spectra nanodiamonds, insert is the zeta potential of nanodiamonds in PBS buffer. Black arrow points to the characteristic dip (1780 cm^−1^) of –COOH group. (**d**) Fluorescence images of HepG2 cells incubated with nanodiamonds and the corresponding (**e**) transmitted images, insert is one enlarged typical cell. (**f**) Quantitative flow-cytometry data showing the inhibition of nanodiamonds’ uptake in the presence of inhibitors for different types of endocytosis, elucidating the cellular uptake mechanisms of nanodiamonds. ***p < 0.001 compared with control (n = 3 for all samples).

In the present study, we used HPHT NDs with nitrogen vacancy (NV) centers, which served as the fluorescent labelling agent. The average size of the NDs was ~100 nm, and their shapes were irregular. Their surfaces were negatively charged and –COOH rich (Fig. 1a–c). The photoluminescence from NDs (red in colour) under green laser excitation was easily found inside cells after their 24 hours incubation in serum-free medium (Fig. 1d). In a typical confocal microscopy image, each cell contained many red dots, suggesting the existence of NDs (Fig. 1d,e). We then investigated the detailed cellular uptake pathway of NDs by pretreating cells with various endocytic inhibitors (each is specific for a particular endocytic pathway) before NDs’ feeding^18^. As shown in Fig. 1f, both NaN_3_ and incubation at low temperature (4 °C) (disturbing the formation of ATP and thus blocking endocytosis^19^) significantly decreased the cellular uptake of NDs by >80%. This indicated that endocytosis of NDs was the major route for their cellular entry. Subsequently, the four different pinocytic pathways were further explored at 37 °C. The results revealed that adding amiloride (a specific inhibitor of the Na^+^/H^+^ exchange required for macropinocytosis^20^) decreased the cellular uptake of NDs by ~50%, while both filipin (inhibiting caveolae-mediated endocytosis through cholesterol sequestration) and monodansylcadaverine (inhibiting formation of clathrin-coated pits) did not significantly altered the cellular uptake amount of NDs. In addition, feature of macropinocytosis (filopodia formation, Supplementary Fig. S1) was also observed in transmitted electron microscopy (TEM) inspections. These results further suggested that macropinocytosis was the major endocytic pathway for NDs.

### The endosomal escape mechanism of NDs

**Figure 2:**
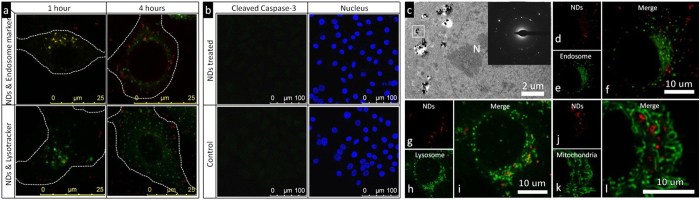
Rapid endosomal escape and subsequent intracellular distribution of nanodiamonds. (**a**) Representative confocal microscopy images showing the fluorescent signals of lysotracker/endosome marker (green) and nanodiamonds (red) in HepG2 cells after cell incubation with nanodiamonds in serum-free medium for 1 or 4 hours. The yellow color indicates the overlap between red (nanodiamonds) and green (endosomes/lysosomes). (**b**) Representative confocal microscopy images showing the intracellular expression of cleaved caspase-3 in HepG2 cells as revealed by their fluorescence intensity (green). The cells treated with normal medium was chosen as control, and the positive control could be found in Supplementary Fig. S2. The nuclei were stained by DAPI (blue). (**c**) TEM images showing the typical intracellular distributions of nanodiamonds in HepG2 cells after their incubation with cells in serum-free medium for 24 hours. (**d**–**f**) Representative confocal microscopy images showing the fluorescent signals of (**d**) nanodiamonds (red) and (**e**) endosome marker (green), and (**f**) the overlap image of (**d**) and (**e**). (**g**–**i**) Representative confocal microscopy images showing the fluorescent signals of (**g**) nanodiamonds (red) and (**h**) lysotracker (green), and (**i**) the overlap image of (**g**) and (**h**). (**j**–**l**) Representative confocal microscopy images showing the fluorescent signals of (**j**) nanodiamonds (red) and (**k**) mitotracker (green), and (**l**) the overlap image of (**j**) and (**k**). (All images were taken from HepG2 cells after their incubation with nanodiamonds in serum-free medium for 24 hours for Fig. 2d–l.)

By checking the time-dependent intracellular trafficking of NDs (Fig. 2a), we found that these particles were mainly trapped in endosomes for a short while (1 hours), and then translocated to cytoplasm shortly afterwards. On the other hand, the NDs were seldom found to co-localize with lysosomes. These results suggested that the NDs escaped from endosomes to cytoplasm shortly after endocytosis, and had little chance to be translocated into lysosomes. This was further confirmed by checking the intracellular cleaved caspase-3 expression, which was an indicator of lysosomal damage^21^,^22^. As shown in Fig. 2b, we found that the NDs treated cells did not show elevated expression of cleaved caspase-3 as compared to that in the control sample (cells treated with normal medium without NDs), indicating that NDs caused little lysosomal damage. The fast endosomal escape was important, as it avoided the NDs-containing endosomes undergo their maturation eventually to lysosomes, while lysosomal damage is known to cause toxicity due to spillage of their inner content^23^. Free from lysosomal damage suggested the low cytotoxicity associated with NDs’ cytosolic release.

We further investigated the intracellular distributions of NDs after endocytosis using TEM, which provided information with excellent spatial resolution. The representative TEM image was shown in Fig. 2c. NDs (dark spots, confirmed by its diffraction pattern as shown in the insert of Fig. 2c) were found to be randomly distributed in the cytoplasm rather than residing inside membrane bound vesicles. To further confirm this, we stained the endosome, lysosome and mitochondria of cells by the respective dyes, and studied the co-localization of NDs with these organelles in live cells using confocal microscopy. As shown in Fig. 2d–l, little fluorescence signal overlapping was observed between NDs and these organelles (endosome (Fig. 2d–f), lysosome (Fig. 2g–i) and mitochondria (Fig. 2j–l)) after 24`hours’ incubation.

**Figure 3:**
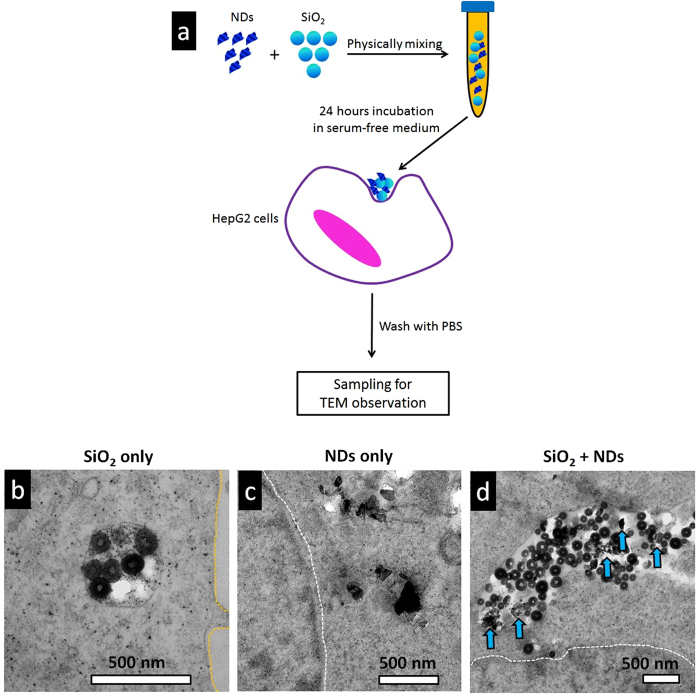
Evidence of nanodiamonds’ rupturing the endosomal compartment, causing their cytosol release. (**a**) Schematic illustration of the experimental design to identify how nanodiamonds escape the endosomal compartments. (**b**) Typical TEM images showing distribution of SiO_2_ in HepG2 cells after cells’ incubation with SiO_2_ only in serum-free medium for 24 hours. (**c**) Typical TEM images showing distribution of nanodiamonds in HepG2 cells after cells’ incubation with nanodiamonds only in serum-free medium for 24 hours. (**d**) Typical TEM images showing distribution of SiO_2_ + NDs in HepG2 cells after cells’ incubation with the mixed nanoparticles in serum-free medium for 24 hours.

To find out how did the NDs escape the endosomal compartments, we designed a co-feeding experiment. We simultaneously fed the cells with NDs and spherical silica NPs, with the latter one known to stay inside the endosomes and evolve with endosomal maturation. Specifically, amorphous spherical SiO_2_ NPs^24^ were physically mixed with the prickly NDs (Supplementary Fig. S3) at weight ratio of 1:1, and then fed to cells for 24 hours (Fig. 3a). We then compared NPs’ intracellular locations of cell samples fed with SiO_2_ only, NDs only, and the mixtures of the two. By observing hundreds of views under TEM for each of the cell samples, we found that most of the SiO_2_ NPs stayed in membrane bounded vesicles (Fig. 3b) for cell samples treated with SiO_2_ NPs only. While NDs were mostly found in cytoplasm for cell samples treated with ND only (Fig. 3c). When the NP mixtures (ND/SiO_2_ with weight ratio of 1/1) were co-fed to cells, we observed that in most cases both SiO_2_ and NDs located in cytoplasm, and it was quite often to find both of them in the vicinity of a partially ruptured vesicle (Fig. 3d). This indicated that the NDs ruptured the membrane of endosomal compartment, enabling the escape of SiO_2_ NPs, which should stay stably in the endosomal compartments otherwise. By examining the intracellular distribution of NDs and/or silica NPs in the respective samples, we gave clear experimental evidence that “rupturing” was the mechanism of NDs’ escaping the endosomes.

### Application of prickly nanodiamonds as vehicles for gene delivery

The unique features of NDs interacting with cells suggested NDs as suitable candidates for delivering macromolecules such as plasmid DNA to cytosol. Herein, we chose green fluorescent protein (GFP) plasmid DNA as a model cargo. Such plasmid DNA was difficult to enter cells due to its negative charge, leading to low DNA expression in the cytosol and thus low green fluorescence^25^,^26^.

We first adsorbed the DNA molecules onto NDs through simple electrostatic attraction (see Methods). In parallel, similar amount of DNA were absorbed onto the spherical SiO_2_ NPs for comparison. Such a sample was chosen for its specific intracellular translocation characteristics being significantly different from that of NDs—upon endocytosis, the SiO_2_ NPs were found to stably reside in endosomes and evolve together with endosomes maturation to lysosomes^27^,^28^. They had fairly high probability of being excreted via exocytosis, but little chance to be released into cytoplasm (Supplementary Fig. S4).

**Figure 4:**
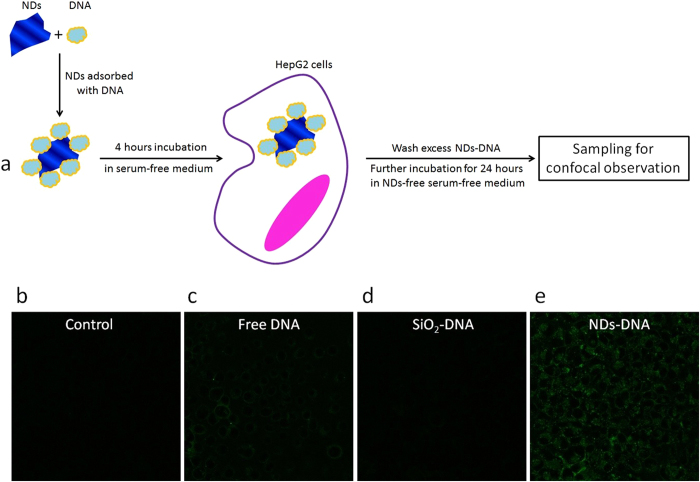
Demonstration of prickly nanodiamonds as vehicles for gene delivery *in vitro*. (**a**) Schematic illustration of the *in vitro* experimental design for gene delivery using nanodiamonds as carrier. (**b**–**e**) typical confocal images of GFP expression in HepG2 cells after cells’ being treated with (**b**) fresh medium, (**c**) free DNA, (**d**) SiO_2_-DNA and (**e**) NDs-DNA.

The *in vitro* transfection experiments were carried out using HepG2 cells with free DNA, SiO_2_-DNA and NDs-DNA (Fig. 4a) at the same DNA feeding concentrations. The feeding concentrations of the SiO_2_ and NDs were also kept the same. It was found that the cellular uptake amount of SiO_2_ NPs was almost twice as that of NDs (Supplementary Fig. S5). Consequently, the DNA uptake amount was higher in SiO_2_ NP-fed cells than in ND-fed ones.

The corresponding expression of cytoplasmic GFP DNA was investigated using confocal microscopy (Fig. 4b–e). Cells treated without DNA were used as control to eliminate the effect of intracellular auto-fluorescence. Comparing to the control sample, negligible amounts of DNA could be observed (Fig. 4c,d) in both the free DNA and SiO_2_-DNA treated samples, while the NDs-DNA treated sample showed significant expression of DNA in cytoplasm of the treated cells (Fig. 4e). The low expression of DNA was expected in the free DNA fed cell samples—the negative charge of DNA molecules made it difficult to enter the cell interior, causing their low expression in the cytosol. In the case of SiO_2_-DNA NPs, despite the fact that the DNA uptake amount in such a sample was higher than that of the NDs-DNA fed ones, the NPs’ incapability of escaping from the endosomal compartments to cytosol obstructed DNA entering the cytoplasm. Therefore, NDs-DNA has the advantages of enhanced cellular uptake and effective endosomal escape, resulting in efficient delivery of DNA into the cell cytoplasm. These findings demonstrated the capability of NDs as an effective intracellular transporter for plasmid DNA.

In the literature, DNA cellular delivery had been attempted using viruses^29^, liposomes^30^ such as Lipofectamine, and various forms of nanoparticles^25^,^31^,^32^. One would care about two aspects when comparing these DNA delivery methodologies. On the one hand, disturbance to normal cell physiology and especially the cytotoxicity of these methodologies is an important measure—one wants to delivery DNA without affecting the normal functions of the cells. In this regard, the viruses approach raises the issues of immunogenicity, carcinogenicity and inflammation, which hinder its potential clinical translation^33^,^34^. The liposome based methods are known to have cytotoxic effect due to the high surface charge required for cellular uptake^35^. For methodologies that employ endocytosis as the cell entry route, i.e., most of the nanoparticle carrier based approaches, the loaded DNA faces the problem of endo-lysosomal confinement and subsequently degradation. A most common strategy to enable the cytosolic release of the carrier vectors is to decorate the vector surfaces with cationic polymer/peptites, which introduce unavoidable cytotoxic effects, depending on their applied size, degrees of branching concentration, etc.^36^. As a comparison, NDs were found to have little cytotoxicity (Supplementary Fig. S6, and Fig. 2b). This property is superior to many other DNA delivery methodologies. On the other hand, the gene transfection efficiency is another important measure. In this regard, we obtained semi-quantitative results of the transfection efficiency of NDs-DNA carriers with comparison made to free DNA (Supplementary Fig. S7).Compared to free DNA, expression of GFP by NDs carrier showed a ~3-fold increase in the green fluorescence intensity, which is proportional to the transfection efficiency of DNA carriers. The improvement is comparable to literature reports using other nanoparticle systems, e.g., gold NPs coated with PEI as nanocarriers for DNA delivery^37^.

## Conclusion

In this study, we found that the internalization of NDs was easily realized via macropinocytosis—a type of nonspecific endocytosis. We uncovered how the prickly NDs escape endosome confinement, i.e., by rupturing the membrane of endosomal compartments shortly after their cellular uptake. Little cytotoxicity was found to associate with the NDs’ intracellular translocation. These features made NDs promising candidates to serve as the carrier for plasmid DNA—both the enhanced cellular uptake (via endocytosis) and the cytosolic release of DNA were found to be critical in enabling the effective delivery of plasmid DNA and its expression. Such carrier platform had a potential to be applied for a general approach to a vast base of cell impermeable molecules, such as siRNA, protein, drug and etc., for effective cytosolic delivery.

## Methods

### Preparation of and characterization NPs

The NDs and SiO_2_ NPs were obtained using our previous published procedures^24^,^28^,^38^. For hollow SiO_2_ NPs used in co-feeding experiments, the as synthesized self-decomposable SiO_2_-NPs^24^ was calcinated to form the required inner hollow structure, being easily distinguishable from other materials with dense TEM contrast inside cells. The morphology and size of the NPs were characterized using low magnification transmission electron microscopy (PhilipsCM120). The surface of NPs was investigated by Fourier Transform Infrared Spectrometer (FTIR; Nicolet 670, Thomas Nicolet, Waltham, MA). The average zeta potential of NDs in PBS buffer solution was measured using a commercial zeta potential spectrometer (ZetaPlus, Brookhaven). The size distributions of NDs in PBS were obtained using dynamic light scattering (DLS). The apparatus used for DLS measurements was an ALV-5000 goniometer (ALV Laser) equipped with a helium–neon laser and a digital correlator. All measurements were carried out at room temperature (25 °C).

### Characterization of NPs interacting with cells

The HepG2 cell line (human liver carcinoma) was employed in this study. The HepG2 cells were cultured in Dulbecco’s modified Eagle’s medium (DMEM, Gibco), supplemented with 10% heat-inactivated Fetal bovine serum (FBS), 2.0 g/L sodium bicarbonate, 0.1 g/L streptomycin sulfate, 0.06 g/L penicillin G and 5.958 g/L HEPES. The feeding concentration of the NPs was always kept at 10 μg/ml unless specified in the corresponding result description. Different cell feeding durations were specified in the corresponding result description.

For TEM study, the cells treated with NPs were fixed, sliced by microtome (Leica, EM UC6) and double stained with aqueous uranyl acetate and lead citrate according to our previous publishedprocedure^28^ before observation under TEM (PhilipsCM120). For all confocal microscopy studies, the cell samples were observed using confocal laser scanning microscopy (TCSP5, Leica) with a 63x water-immersion objective lens.

The detailed cellular uptake pathway of NDs was investigated by pretreating cells with various inhibitorsat 4 °C for 3 hours in serum-free medium. Then the original medium was discarded, and followed by incubating with NDs for 6 hours in serum-free medium. Finally, the cells were washed with PBS, fixed with 70% ethanol, and then processed for flow-cytometry (FACScan, Becton Dickinson, Canada) analysis. All data were shown as mean ± SD (from three independent experiments) and significantly different (p < 0.05) from control (analyzed by Student’s t test). The intracellular localizations of NDs were also investigated by immunofluorescence, i.e., staining cell samples with the endosome marker (C10586, Invitrogen), LysoTracker (L7526, Invitrogen) and MitoTracker (M7514, Invitrogen) according to the manufacturer’s instructions.

### *In vitro* gene delivery using NPs as carrier

The NPs were firstly functionalized with –NH_2_ to have slightly positive charge surface for absorbing the negative charged plasmid DNA (Supplementary Fig. S8). Briefly, 1 mg NDs (or SiO_2_ NPs) were firstly dissolved in 1 ml mixture of H_2_O and ethanol (1:1 in volume), then 10 μl (3-Aminopropyl)triethoxysilane (APTES) was added. The whole mixture was put in the ultra-sonication bath for 2 hours at room temperature. Finally, the NPs were washed with deionized water for several rounds, and dispersed in 1 ml deionized water before use. Supercoiled plasmid DNA pEGFP-N1 (Clonetech) carrying the EGFP gene was harvested from transformed E.coli of DH5α (Invitrogen) strain shaken overnight in LB broth supplemented with 100 μg/ml of ampicillin (11259, USB), followed by extraction with Purelink HiPure Plasmid Midiprep kit (Invitrogen). The adsorption of DNA onto the NPs was done by adding fresh prepared DNA to NPs solution and putting in fridge at 4 °C overnight. Then the mixture was centrifuged and washed with deionized water for several rounds, the amount of adsorbed DNA was calculated from the amount of DNA before and after adsorption by NanoDrop 2000c Spectrophotometer (Thermo). 7 μg DNA was found to absorbed on 1 mg SiO_2_ NPs while 3.7 μg DNA was found to adsorbed on 1 mg NDs. We fed HepG2 cells with 100 μg/ml SiO_2_-DNA or NDs-DNA for 4 hours in serum-free medium, then changed into fresh full medium for another 24 hours incubation before observation under confocal microscopy.

## Additional Information

**How to cite this article**: Chu, Z. *et al.* Rapid endosomal escape of prickly nanodiamonds: implications for gene delivery. *Sci. Rep.* **5**, 11661; doi: 10.1038/srep11661 (2015).

## Supplementary Material

Supplementary Information
