# Supplementary material for: Rapid endosomal escape of prickly nanodiamonds: implications for gene delivery
Source: Sci Rep. 2015 Jun 30;5:11661. doi: 10.1038/srep11661 (PMC4485068; doi:10.1038/srep11661)
Supplement: Supplementary Information [file srep11661-s1.pdf]

Supporting Information

Rapid endosomal escape of prickly nanodiamonds: implications for gene delivery

Zhiqin Chu[1,2], Kaikei Miu[3], Pingsai Lung[1], Silu Zhang[1], Saisai Zhao[1], Huan-Cheng Chang[4], Ge Lin[3], Quan Li[1,5,*]

1. Department of Physics, The Chinese University of Hong Kong, Shatin, New Territories, Hong Kong
2. 3rd Institute of Physics, University of Stuttgart, 70569 Stuttgart, Germany
3. School of Biomedical Sciences, Faculty of Medicine, The Chinese University of Hong Kong, Shatin, New Territories, Hong Kong
4. Institute of Atomic and Molecular Sciences, Academia Sinica, Taipei 106, Taiwan
5. The Chinese University of Hong Kong ShenZhen Research Institute, ShenZhen, China

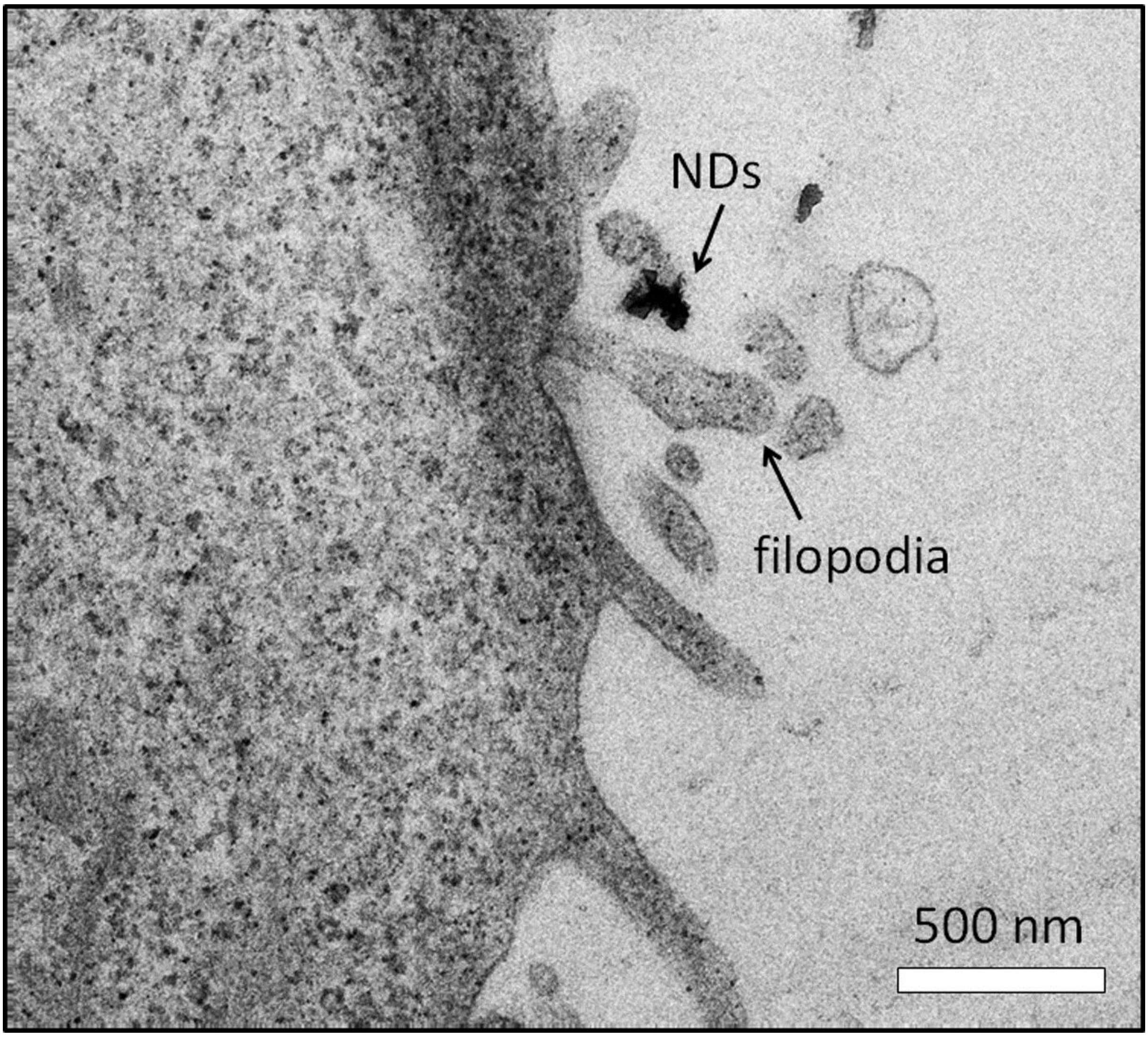

Figure S1. TEM image showing the formation of filopodia (a feature of macropinocytosis) in nanodiamonds' treated cells. HepG2 cells were treated with NDs in serum-free medium for 24 hours. The arrows were pointed to NDs and filopodia in the image.

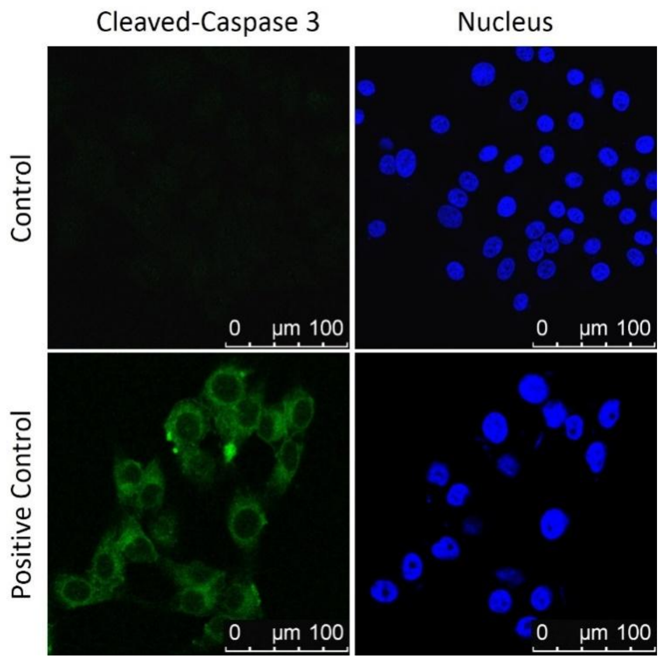

Figure S2. Representative confocal microscopy images showing the intracellular expression of cleaved caspase-3 in HepG2 cells as revealed by the fluorescence of cleaved caspase-3 (green). The nuclei were stained by DAPI (blue). The cells treated with doxorubicin were chosen as positive control. The cells treated with medium only were chosen as control.

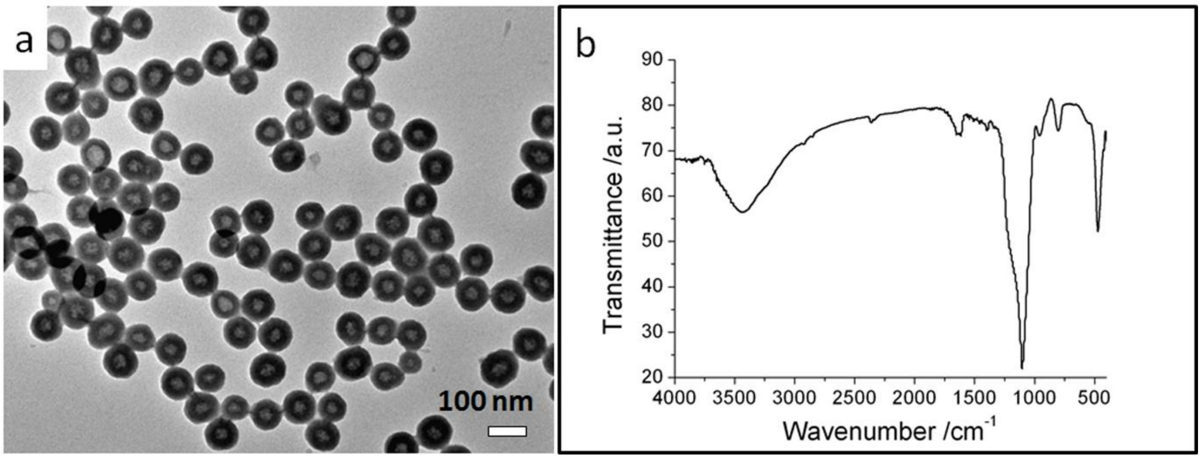

Figure S3. Characterizations of the hollow spherical SiO_2 NPs. (a) TEM image of hollow SiO_2 NPs (~90 nm in diameter). (b) FTIR spectrum taken from hollow SiO_2 NPs showing the typical surface chemistry of SiO_2.

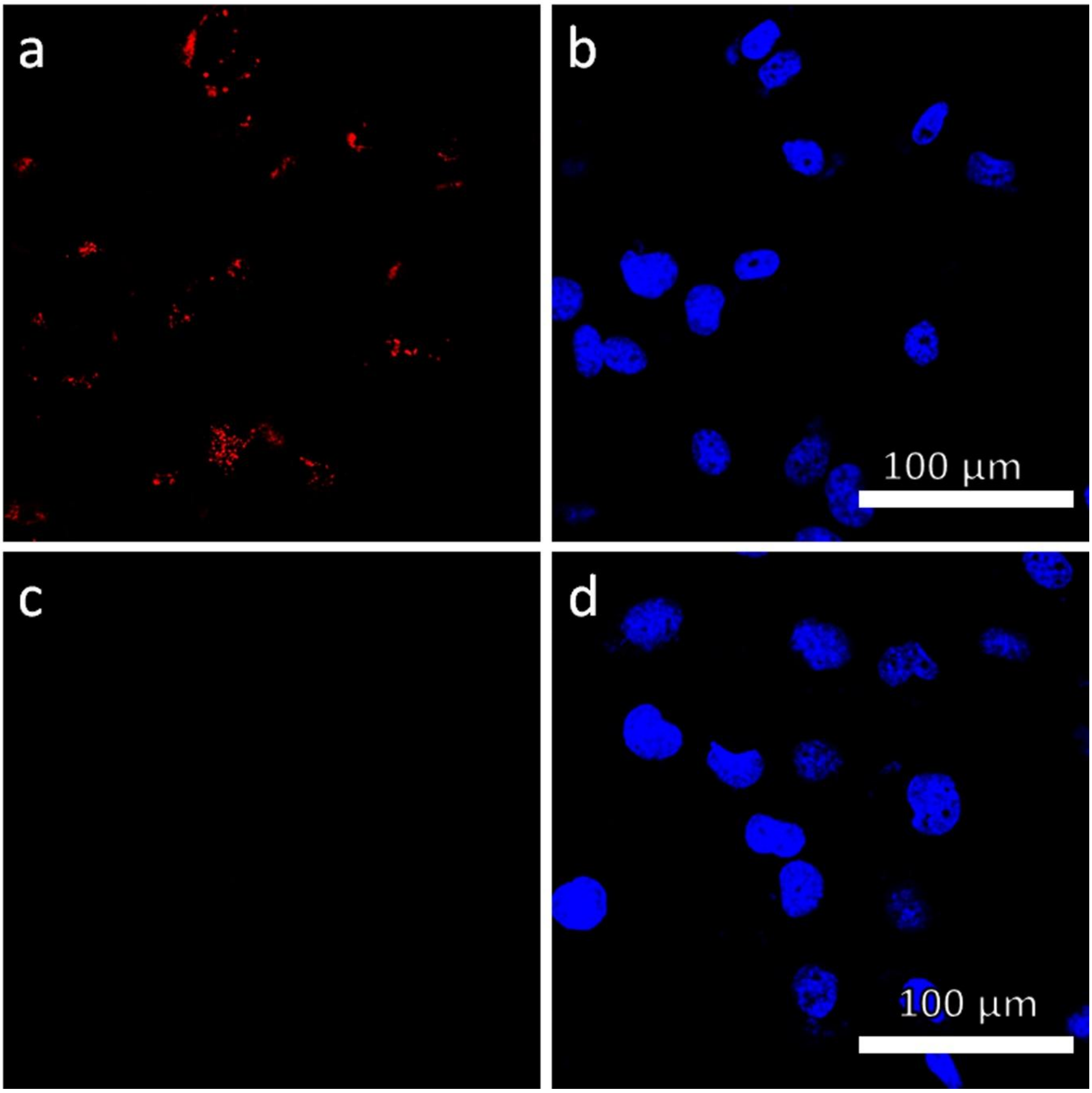

Figure S4. Representative confocal microscopy images showing the excretion of 50 nm fluorescent amorphous silica nanoparticles in HepG2 cells as revealed by the fluorescence of silica nanoparticles (red). The nuclei were stained by DAPI (blue). (a,b) HepG2 cells were incubated for 12 hours in serum-free medium with silica nanoparticles. (c,d) HepG2 cells were incubated for additional 12 hours in nanoparticle-free serum-free medium. Greatly reduced fluorescent signal from the silica nanoparticles (red dots) shows most of these nanoparticles exited the cells after the ‘‘washing’’ process.

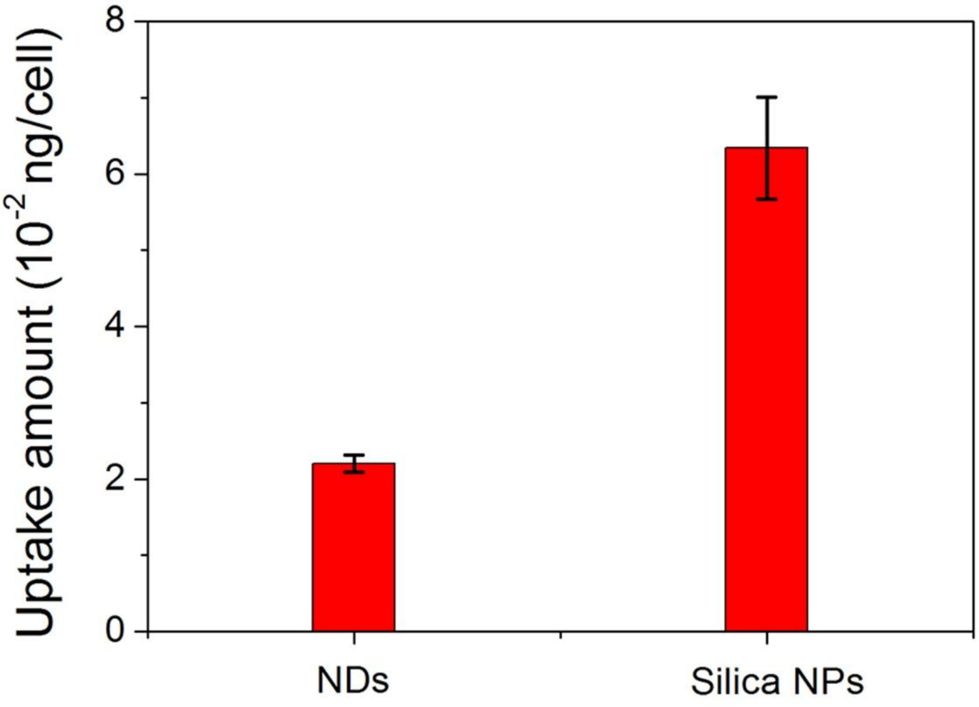

Figure S5. Cellular uptake amount of prickly NDs and silica nanoparticles in HepG2 cells. The HepG2 cells were incubated with various samples at 10 µg/ml for 24 hours in serum-free medium. At the end of incubation, the cells were washed, collected and dissolved using 10% sodium dodecyl sulfate (SDS) for further analysis. The amount of NDs were determined by its photoluminescence, while the amount of silica NPs were determined by inductively coupled plasma atomic emission spectroscopy (ICP-AES).

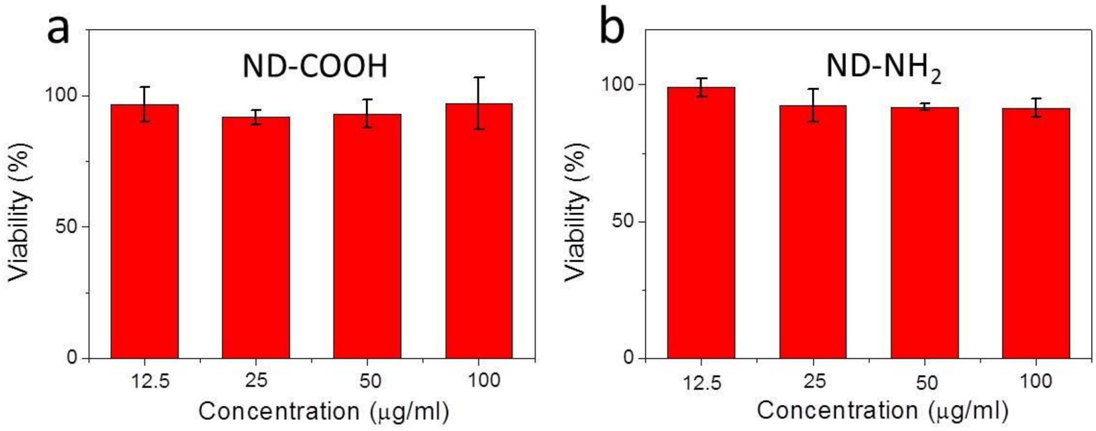

Figure S6. Cytotoxicity of NDs on HepG2 cells determined by MTT assay. The cells incubated with (a) ND-COOH or (b) ND-NH_2 particles for 4 hours in serum free medium. Data are presented with mean $\pm$ standard deviation (SD) from three independent experiments. Significance indicated by $p < 0.05$, analyzed by student's t test.

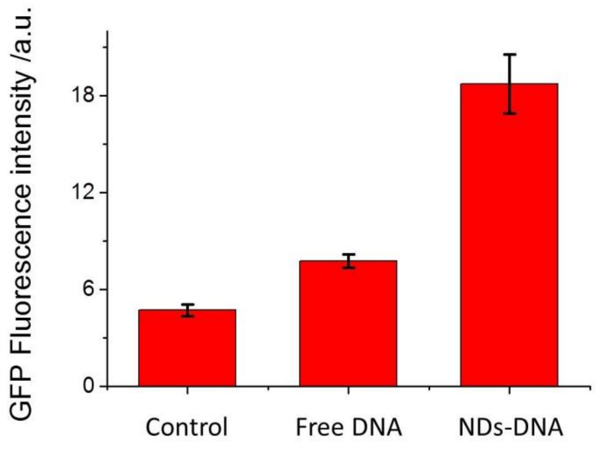

Figure S7. Transfection efficiency of plasmid DNA loaded in various carriers. The amount of intracellular expressed GFP was semi-quantitatively estimated from the corresponding fluorescence intensity of confocal images (Fig. 4 in main text).

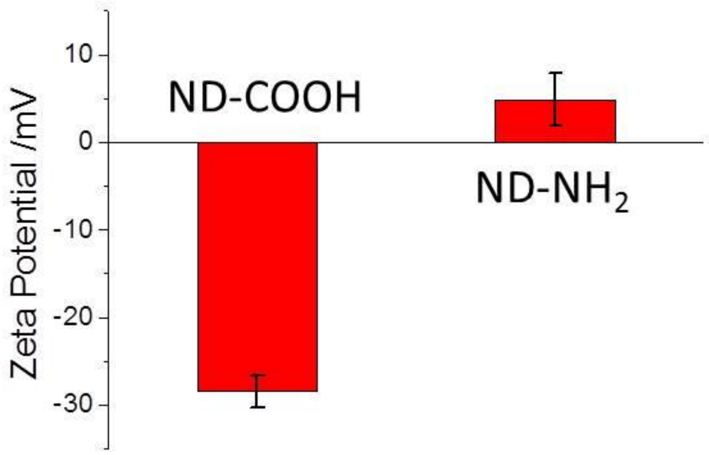

Figure S8. Zeta potential of ND-COOH and ND-NH_2 particles (designed for adsorbing plasmid DNA) dispersed in PBS buffer.
